# Evolutionary Models for Formation of Network Motifs and Modularity in the *Saccharomyces* Transcription Factor Network

**DOI:** 10.1371/journal.pcbi.0030198

**Published:** 2007-10-26

**Authors:** Jonathan J Ward, Janet M Thornton

**Affiliations:** 1 European Bioinformatics Institute, Wellcome Trust Genome Campus, Hinxton, Cambridge, United Kingdom; 2 European Molecular Biology Laboratory (EMBL), Heidelberg, Germany; Harvard University, United States of America

## Abstract

Many natural and artificial networks contain overrepresented subgraphs, which have been termed network motifs. In this article, we investigate the processes that led to the formation of the two most common network motifs in eukaryote transcription factor networks: the bi-fan motif and the feed-forward loop. Around 100 million y ago, the common ancestor of the *Saccharomyces* clade underwent a whole-genome duplication event. The simultaneous duplication of the genes created by this event enabled the origin of many network motifs to be established. The data suggest that there are two primary mechanisms that are involved in motif formation. The first mechanism, enabled by the substantial plasticity in promoter regions, is rewiring of connections as a result of positive environmental selection. The second is duplication of transcription factors, which is also shown to be involved in the formation of intermediate-scale network modularity. These two evolutionary processes are complementary, with the pre-existence of network motifs enabling duplicated transcription factors to bind different targets despite structural constraints on their DNA-binding specificities. This process may facilitate the creation of novel expression states and the increases in regulatory complexity associated with higher eukaryotes.

## Author Summary

Networks are a simple and general way of representing natural phenomena that range in scale from the social interactions between people to the organization of circuits on a microchip. Many networks have been found to contain repeated patterns of connections between small groups of nodes. These patterns, termed network motifs, are thought to be involved in controlling the flow of information through the network. This article investigates the processes that led to the formation of the two most common types of motif in the network controlling gene expression in baker's yeast. Around 100 million y ago, yeast's ancestor underwent a whole-genome duplication, which resulted in the organism containing four copies of each gene rather than the usual two. The duplicated genes that remain in the yeast genome are used to infer the two mechanisms that give rise to network motifs. These are rewiring of interactions between genes, and the duplication of proteins that control gene expression (transcription factors). These two processes are complementary with the rewiring mechanism enabling duplicated transcription factors to regulate the expression of different genes. It appears likely that these two processes are involved in enabling the increases in complexity that are associated with multicellular life.

## Introduction

One of the most fundamental questions in biology is how incremental evolutionary changes lead to the observed complexity in biological systems. The advent of genome sequencing and associated functional genomic technologies have provided the first evidence for the origins of complexity on an organism-wide scale. Modularity is an emergent property of biological networks that has been observed in metabolic [1], protein–protein interaction [2], and transcription factor networks (TFNs) [3]. Several explanations have been put forward for the evolution of modular biological systems, which include robustness to mutational [4] and environmental perturbations [5], insulation against cross-reactivity between alternative signalling cascades [6], and selection for survival in multiple environments [7].

Parallel studies of small, artificial TFNs have demonstrated that alterations in network topology and components can be used to create a wide range of dynamic properties such as bistability and oscillations. However, relatively few local topologies are widely observed in natural networks [3,8]. For example, although a circuit composed of two inhibitory transcription factors (TFs) arranged in a feedback loop has been shown to act as a stable memory element in the lambda phage virus and artificial systems [9], this topology is uncommon in both the Escherichia coli and Saccharomyces cerevisiae transcriptional networks so far uncovered [3,8]. An outstanding question is whether the absence of these and other local topologies is a result of mechanistic or functional constraints on network evolution.

**Figure 1:**
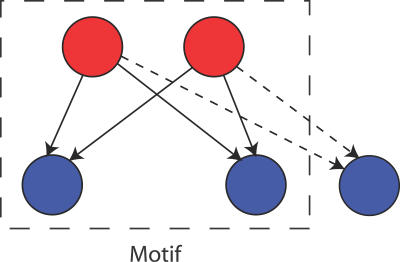
The Bi-Fan Motif and Its Extension to Bi-Fan Arrays Genes that encode TFs are coloured red, and genes that do not encode TFs are coloured blue. Accumulation of *n* common target genes (additional connections are represented by dotted lines) leads to formation of a bi-fan array containing *n*(*n* − 1) / 2 bi-fan motifs.

In this article, transcription regulatory interactions in the yeast S. cerevisiae were defined using the large-scale chromatin immunoprecipitation (ChIP-on-chip) dataset of Harbison et al. [10] These interactions were used to define a network with nodes representing genes and directed edges binding of a protein encoded by a TF gene to the promoter of a target gene. We begin by investigating several growth models for the formation of bi-fan motifs, which involve a pair of TFs that bind the promoters of two target genes, as shown in Figure 1. The bi-fan motif is typically embedded in extended structures that we term the bi-fan array, involving a pair of TFs that both regulate a larger number of common target genes. Figure 1 illustrates how the number of bi-fan motifs within an array grows quadratically as target genes are added. In later sections, we demonstrate a specific structural relationship between bi-fan arrays and the feed-forward loop (FFL) motif, and a common origin for many of these network structures.

The topology of the bi-fan motif suggests several evolutionary mechanisms for its formation, including duplication of either TFs or target genes [11]. It is also possible that the motifs could have arisen from rewiring of regulatory interactions as a result of *cis-*sequence evolution in genic promoter regions or the *trans-*evolution of the protein sequences encoding TFs. The *cis*-sequence evolution refers to mutations in noncoding regions that alter the binding affinity of TFs for a particular promoter, thus affecting the expression of genes in close proximity on the chromosome [12,13]. Conversely, *trans*-evolution typically involves mutations in the sequences encoding TFs that alter, for example, their DNA-binding specificity. These *trans*-changes have the potential to alter the expression of large numbers of genes [12,13]. In this article, the relative contributions of these mechanisms are investigated by defining a common evolutionary origin for pairs of genes using the whole-genome duplication (WGD) event that occurred in S. cerevisiae after its divergence from Kluyveromyces waltii [14,15].

## Results

### Bi-Fan Motifs Are Organised in Arrays

We investigated the organisation of bi-fan motifs in the yeast TFN using two algorithms that have been used previously for detecting motifs in directed networks [3,8]. These algorithms fix both the in-degree and out-degree of each node and then randomly replace the edges in the network. This approach can then be used to detect motifs that occur more frequently in the native network than a large ensemble of random networks (see Methods for further details). Although the original methods for detecting network motifs involved exhaustive enumeration of all small (typically 2- to 6-node) subgraphs in the network, previous work [3,16] suggests that bi-fan motifs are embedded in larger structures within the yeast and E. coli TFNs. In fact, it is possible to show (see Methods for details) that the overrepresentation of bi-fan motifs in any directed network is associated with the array structures shown in Figure 1.

Bi-fan arrays were identified in the yeast TFN by searching for pairs of TFs with a number of shared targets that exceeded the number found in the randomized networks with *p* < 10^−4^. A description of the *p*-value calculation is included in the Methods section. A total of 442 bi-fan arrays were identified at this strict significance threshold. These arrays account for a total of 1.25 × 10^5^ (68% of the total) bi-fan motifs compared with an expected number of 7.3 × 10^3^ under the null model. The overrepresentation of bi-fan motifs in the *Saccharomyces* TFN (shown in Table 1) can therefore be attributed to a relatively small number of bi-fan arrays that, on average, regulate a large number of target genes. The following two sections investigate the influence of gene duplication on formation of the bi-fan array structure.

**Table 1:**
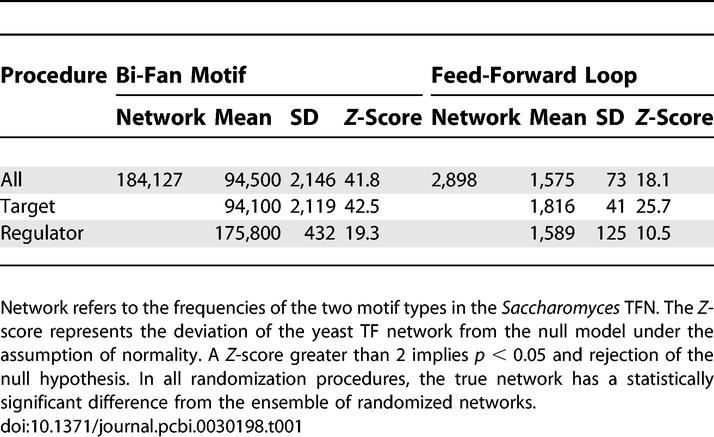
Summary of Statistical Significance of Network Motifs under Several Randomization Procedures

### Effect of Gene Duplication on the Formation of Bi-Fan Arrays

Two approaches were used to identify genes that have arisen from duplication. The first method involves using genes that were created from the most recent WGD in the evolution of S. cerevisiae [14,15]. These data are likely to be of very high fidelity because of the requirement for genes to reside in regions of doubly conserved synteny with the K. waltii genome [15]. Another advantage of defining common origin using WGD data is that duplication of all genes occurred simultaneously, and duplicates initially possessed very similar promoter regions. This provides a means to estimate the relative *cis-* and *trans*-conservation rates upon gene duplication, as shown in Table 2.

**Table 2:**
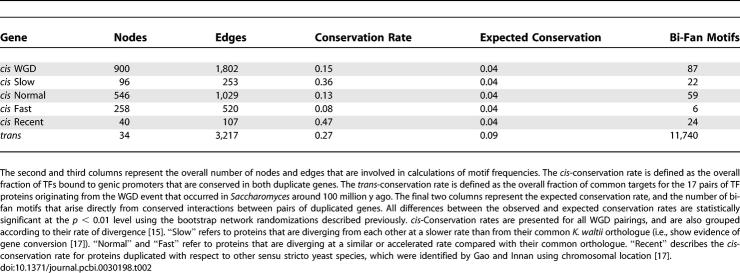
Network Properties of Genes Originating from Duplication

Table 2 shows that the *trans*-conservation rate is relatively high, which is caused by nine of the 17 WGD duplicates forming statistically significant bi-fan arrays. These arrays contain a substantial proportion of the network's bi-fan motifs. Conversely, the *cis*-conservation rate for all promoters duplicated by WGD is low, with relatively few bi-fan motifs arising from conserved interactions. In the case of promoters of genes that are diverging rapidly, the conservation rate is only slightly above that expected for randomly selected promoters and indicates substantial plasticity in promoter binding.

It is also possible to rule out more recent single-gene duplications as a significant source for bi-fan motifs, as these have been estimated to occur very infrequently in *S. cerevisiae,* at a rate *λ* = 1–6 × 10^−5^ per gene per million y [17]. An upper bound for the number of single-gene duplications that have occurred since the divergence of S. cerevisiae from K. waltii can be calculated by assuming that the rate of duplication is at the upper limit and that the rate of loss is zero. The number of gene duplications is then given by the exponential growth model

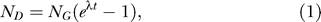
where *N_G_* = 3,500 is the approximate number of single-copy genes in *S. cerevisiae,* and *T* = 100–150 million y is the time since WGD [17]. Equation 1 suggests that the number of single-gene duplications that have occurred since WGD, *N_G_*, is less than 35. Conservation at the levels shown in Table 2 would not result in a large number of bi-fan motifs originating from target gene duplication.

### Effects of Ancient Gene Duplication Events

WGD is a feature in the evolution of most known eukaryote organisms, including chordates [18]. However, fewer than 10% of yeast proteins originated from the latest WGD in the *Saccharomyces* lineage. More ancient gene duplications account for the majority (90%) of proteins encoded in the yeast genome [19]. For this reason, we identified duplicates with a more ancient common origin using domain assignments from the Pfam HMM library [20] (see Methods for further details). The results shown in Table 2 have demonstrated that the promoter-binding patterns of duplicate target genes are likely to have diverged on time-scales longer than 100–150 million y, so the analysis is restricted to TFs with common origin identified with the structure of their DNA-binding domains. These results indicate that a total of 27 bi-fan arrays involve TFs with structurally similar DNA-binding domains, accounting for a total of 14.4% of the bi-fan motifs. 239 bi-fan arrays containing 49.2% of the motifs involve two nonhomologous TFs with the remainder involving at least one TF with an unknown structure. This suggests that more ancient TF duplications have also contributed to the formation of bi-fan motifs in the network (see Figure S1).

**Figure 2:**
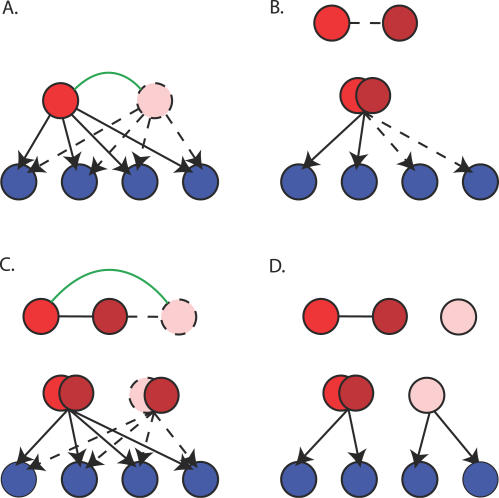
Growth Models for Formation of Bi-Fan Arrays Nodes originating from gene duplication are connected by green undirected edges. Black undirected edges represent protein–protein interactions, and dotted edges/nodes represent network components gained with respect to time. (A) Simple gene duplication scenario results in formation of bi-fan array regulated by homologous TFs. (B) Nonhomologous TFs form bi-fan arrays by accumulation of common target genes. A low-affinity protein–protein interaction between the TFs allows combinatorial control of targets. (C) Duplication of one component of a regulatory complex leads to creation of two regulatory complexes. Conservation of protein–protein interactions creates nonhomologous bi-fan arrays originating from gene duplication. (D) The loss of one of the protein–protein interactions between duplicated TFs and their common binding partner in (C) has the potential to create binding diversity in homologous TFs.

In summary, the redundancy of duplicated TFs results in the formation of bi-fan arrays, although the majority of these network structures do not arise directly from gene duplication. Conversely, the duplication of target genes does not appear to contribute greatly to formation of bi-fan arrays because the network is subject to greater *cis*-plasticity. This difference also arises from the different statistical properties of the (compact) in-degree distribution and the (power-law) out-degree distributions [21]. Taken together, these results suggest that the two major processes that contribute to the formation of bi-fan motifs are duplication of TFs and the accumulation of common target genes, as depicted in Figure 2A–2B.

The colocalization of nonhomologous TFs at genic promoters is likely to involve a combination of two physical mechanisms. The first mechanism involves the presence of binding sites for the two TFs that occur independently in the same set of genic promoters [22]. This process could also enable cooperative binding if a TF displaces nucleosomes that occlude the binding site of a second TF [23]. The plasticity in the promoters of duplicated genes, shown in Table 2, suggests that bi-fan arrays could have arisen from mutations in promoter regions and subsequent selection for TF binding at numerous dispersed loci. The second mechanism involves protein interactions between the TFs that enable cooperative binding to DNA. For example, mitogen-activated protein kinases without intrinsic DNA-binding affinity are localised to actively transcribed genes during the stress response in yeast via interactions with other proteins [24]. It has also been shown previously [8] that protein–protein interactions tend to occur between pairs of TFs that form bi-fan motifs, and we have confirmed that this property also applies to the bi-fan array structure (Figure S1). In the following section, we investigate how gain and loss of protein–protein interactions could cause duplicated TFs with similar DNA-binding specificities to bind different targets in vivo.

### Higher-Order Effects of TF Duplication and the Generation of Novel Expression States

The existence of bi-fan arrays involving nonhomologous TFs suggests that TF duplication could also increase the frequency of these network features. For example, duplication of a TF that forms a regulatory complex would create two further bi-fan arrays, as depicted in Figure 2C. These network features appear as triplets of TFs that form bi-fan arrays with each other, and where two members of the triplet are related by WGD. The network includes 39 of these triplets, containing a total of 2.47 × 10^4^ bi-fan motifs.

The statistical significance of the triplets of bi-fan arrays involving a pair of TFs originating from WGD can be computed by constructing a null model where the 442 bi-fan arrays are fixed and the 17 WGD relationships are added randomly to the network. This approach can then be used to compare the frequency of these network topologies to that in a large number of randomized networks. The expected number of triplets in the random model is 2.96 with *p* < 10^−6^, demonstrating that these network features are a statistically significant property of the network. Further details are provided in Figure S2. Since the WGD duplications occurred simultaneously [14], can be identified with high confidence [15], and were not succeeded by a large number of subsequent duplications [17], it is possible to assign half of the bi-fan motifs in these arrays to *trans*-regulatory interactions that were conserved after gene duplication. This accounts for a further 9.9% of the bi-fan motifs, and suggests that almost one-fifth of the motifs in the 442 bi-fan arrays can be attributed to a single WGD event.

A notable feature of the TFs duplicated by WGD is their very similar consensus DNA-binding specificities. Examples include the TFs *MSN2p* and *MSN4p,* which bind the stress response element AGGGG [25] and the leucine zippers *YAP1p* and *YAP2p,* which both bind the canonical sequence TTAGTCAGC. These are not isolated examples; almost all pairs of TFs that originate from WGD have similar DNA-binding motifs where these are known [10]. It is therefore not surprising that binding cross-reactivity causes duplicated TFs to occupy similar sets of promoters with the associated conservation of common bi-fan arrays. A more pertinent question is therefore which physical mechanisms enable these TFs to bind different targets in vivo.

The most likely mechanism for the divergence of promoter occupancy is that one of the duplicated TFs binds DNA cooperatively with another TF or cofactor via protein–protein interactions [26] or the modification of chromatin structure [23]. The second TF, which lacks such an interaction, cannot bind these promoters with high affinity. A specific example is provided by the forkhead TFs *FKH1p* and *FKH2p,* which bind overlapping sets of promoters and have identical DNA-binding preferences in vitro. It has been shown experimentally that differential promoter occupancy is achieved in vivo by *FKH2p* binding DNA cooperatively with the second TF, *MCM1p* [27]. This process is recapitulated by our analysis, which indicates that *FKH2p* forms a bi-fan array with *MCM1p,* but that this interaction is not shared by *FKH1p.* Our analysis also implicates the cell-cycle regulator *SWI6p* as being involved in creating the differential promoter occupancy between the two forkhead TFs.

The processes by which the TFs diverge in promoter binding propensities can be understood in terms of conventional models for the functional divergence of gene duplicates [28,29]. Immediately after duplication, the derived TFs are involved in an identical set of bi-fan arrays to the ancestral TF. The gain of an interaction that enables cooperative DNA-binding in one member of the pair is known as neofunctionalization, with subfunctionalization involving the loss of such interactions, depicted in Figure 2D. Of the two mechanisms for functional divergence, subfunctionalization is likely to be the dominant source of binding diversity, since the loss of a protein interaction may involve only a few degenerative mutations in one of the TFs, whereas gain requires formation of a novel interaction and subsequent accumulation of target genes [28–30]. This is supported by the rates of sequence evolution [15] in duplicated TFs. In the two pairs of whole-genome–duplicated TFs that have accelerated evolutionary rates compared with their K. waltii orthologue (the cell-cycle regulators *FKH1p* and *FKH2p,* and the stress response genes *SKN7p* and *HMS2p*), the faster-evolving proteins are involved in bi-fan arrays with fewer partner TFs than the more slowly evolving paralogue (see Table S3).

In summary, many bi-fan motifs in the *Saccharomyces* TFN originate from WGD. We have provided evidence that the functional divergence of duplicated TFs, which is likely to be involved in the generation of novel expression states, can be understood in terms of the patterns of gain and loss of bi-fan motifs within the overall structure of the network. The following section investigates the influence of WGD on the formation of FFL motifs.

### FFL Motifs Are Formed by Elaborations on Bi-Fan Arrays

**Figure 3:**
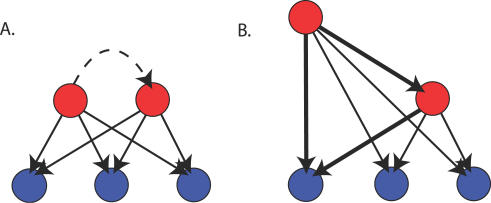
Formation of FFLs (A) Bi-fan array containing three individual bi-fan motifs. (B) Formation of a regulatory interaction between the two transcription regulators generates a feed-forward array containing three FFL motifs (a single example is highlighted in bold).

Having suggested putative evolutionary models for the formation of bi-fan motifs in the S. cerevisiae TFN, we now turn our attention to the FFL. Although the FFL has a topology that appears distinct from the bi-fan motif, the presence of bi-fan arrays suggests another simple mechanism for formation of large numbers of FFL motifs. This process is depicted in Figure 3. In total, there are 43 stastically significant bi-fan arrays that form at least one regulator–regulator interaction, accounting for a total of 1,773 (61.2% of the total) FFL motifs in the TFN. Since these pairs of transcription regulators are expected to be involved in only 36 FFLs, these network features are sufficient to explain the deviation from the null model. The yeast WGD data indicate that four FFL arrays arise directly from WGD containing 334 (18.8%) FFL motifs. A further 11 FFL arrays, containing 299 (16.8%) FFL motifs, involved one of the bi-fan arrays conserved after TF duplication. In none of these cases were the FFL-forming interactions conserved between duplicated TFs.

**Figure 4:**
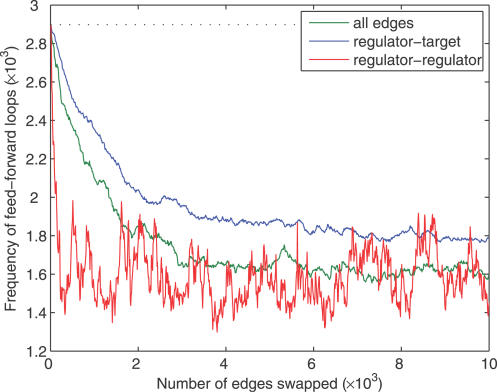
Frequency of FFL Motif under Different Randomization Conditions The curves were generated by starting with the observed network and swapping randomly selected pairs of edges. The green curve corresponds to randomization of all edges, the red curve includes randomization of regulatory connections between TFs, and the blue curve randomization of edges between TFs and genes with *k_out_* = 0, which are referred to as target genes.

**Figure 5:**
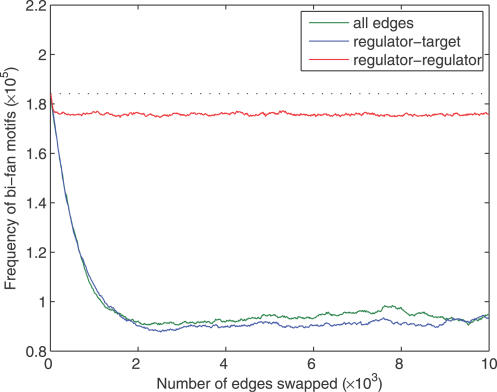
Frequency of Bi-Fan Motifs in the TFN under Different Randomization Conditions

We investigated whether FFLs were a statistically significant feature of the network given its bi-fan structure by randomizing edges between transcription regulators while holding interactions between transcription regulators and nonregulators constant (see Methods). This procedure fixes the vast majority of edges present in bi-fan arrays but involves rewiring of the regulatory interactions between TFs that could give rise to FFLs. Table 1 and Figure 4 show that the FFL topology remains statistically significant under this null model. Figures 4 and 5 show the frequencies of FFLs and bi-fan motifs as pairs of directed edges are swapped randomly, and demonstrate the sensitivity of the number of FFLs to rewiring of a small number of regulator–regulator interactions. Figure 5 confirms that the number of bi-fan motifs is affected only weakly by randomization of interactions between transcription regulators.

The majority of FFL motifs in the yeast TFN result from one or two direct regulatory interactions existing between TFs that form a statistically significant bi-fan array. Although experiments involving randomization of edges between TFs while other parts of the network are fixed suggest that the FFL motif remains overrepresented in natural networks, independently of the presence of bi-fan arrays, it is also possible that the FFL-forming edges could arise from some other nonselective process such as gene duplication. To investigate this question, we used a generalized linear model [31] to fit the probability of a directed regulatory interaction between TF, *a*, and a second TF, *b*, as a function of several local network properties (see Methods for full list). This statistical model was used to identify the network variables that are informative in predicting whether such an interaction occurs.

**Figure 6:**
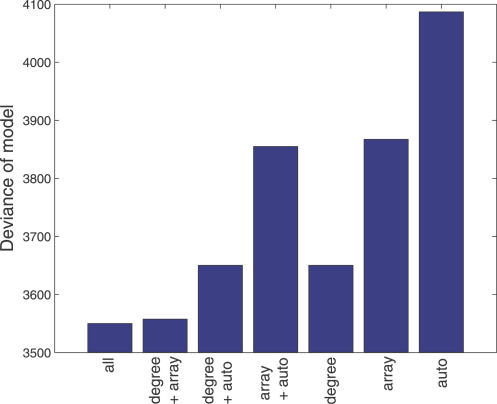
Error of Generalized Linear Models When Used to Fit the Probability of a Transcriptional Regulatory Interaction between a TF and a Second TF “all” refers to models trained on the out-degree, the number of targets shared by the pair of TFs (“array”), and a binary variable representing the presence of an autoregulatory interaction in the target TF (“auto”). A comparison is shown with linear models trained on subsets of these three variables.

The final model indicates that the probability of forming a regulatory interaction increases with the out-degree of node *a* and the number of targets shared by the pair of TFs (i.e., the size of the bi-fan array), but that interactions are suppressed if the second TF *b* directly (auto-) regulates its own transcription. Figure 6 shows a measure of the error of optimized linear models involving subsets of these variables, and indicates that the out-degree has the greatest influence on the probability of forming a regulator–regulator interaction. This would be expected under a neutral model; however, the importance of the second term indicates that there is a propensity toward formation of FFLs from bi-fan arrays in the yeast TFN. This supports there being positive selection toward formation of the FFL motif and the signal-processing properties associated with this topology [32].

### Contribution of Duplicative Bi-Fan Arrays to the Formation of Modular Network Structures

The previous sections have demonstrated that network motifs are typically organized in larger structures that are likely to have originated from two specific growth models. In this section, we investigate whether network motifs originating from duplication of TFs also contribute to more global properties of the network such as its overall modularity [33]. This feature of the TFN was investigated by using a divisive algorithm for partitioning the network into densely connected groups of nodes, which constitute modules, with sparser connections between groups [34]. The network was partitioned into 18 modules with an overall modularity score *Q* = 0.50, which suggests significant community structure [33].

**Figure 7:**
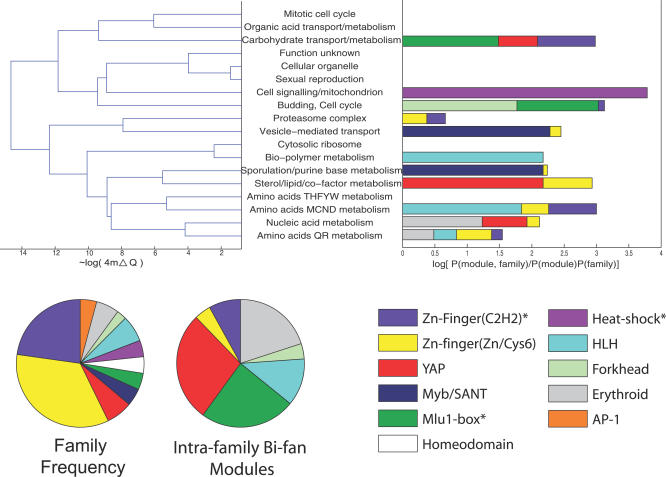
Tree Representation of the Division Path of the Network Clustering Algorithm, and Enrichment of TF Families within Each Module Enrichment is calculated using log-odds and includes families with more than two members in each module. The pie charts show the frequencies of the major families of transcription regulator in the Harbison et al. dataset and the number of homologous bi-fan motifs involving members of these families. Asterisks denote families that have statistically significant common module membership at the *p* < 0.05 level under a permutation test.

The dendrogram in Figure 7 shows a representation of the division path of the algorithm and enriched functional annotations associated with all genes in the extant modules (see Text S1). The algorithm defines a hierarchy of modular structures, with the more “coarse-grained” solutions also representing relevant network structures [34]. In this case, the five coarsest granularity partitions represent the broad functional classes of small molecule transport, cell cycle/reproduction, protein synthesis, protein degradation, and metabolism. Figure 7 also shows enrichment of structural families within each module, and indicates that members from several structural families of DNA-binding protein are not distributed uniformly.

The most recent WGD in *Saccharomyces* can be used to investigate whether duplicated TFs diverge from the ancestral network module, and whether the duplication has contributed to the overall modularity of the network. This latter property is quantified by calculating the change in the modularity upon deletion of each node, which allows identification of modular (Δ*Q* > 0) and nonmodular TFs. Of the 15 pairs of TFs where both members bind a significant number of promoters under the conditions assayed by Harbison et al., 11 are members of the same module (*p* < 0.01 under permutation of module labels). In nine of the pairings, both TFs contribute positively to the modularity of the network, suggesting that gene duplication is involved in the formation of modular networks (the scores are tabulated in Table S3).

There are three further pairs of duplicated TFs in which the sign of Δ*Q* differs between the duplicates, and in which the membership of bi-fan arrays has diverged asymmetrically. If subfunctionalization, which in this context involves the loss of common bi-fan arrays, is the dominant source of functional divergence [30], these examples suggest that the TF that retains the majority of the ancestral functions remains a global (nonmodular) regulator, and that the mutations lead to specialization of its duplicate. Interactions between TFs that lead to creation of FFL arrays also tend to increase network modularity, since the majority (31 out of 43) involve intramodule connections (*p* < 0.01).

## Discussion

We have shown that the overrepresentation of bi-fan motifs in any directed network is associated with bi-fan array structures rather than individual network subgraphs. This property has been observed empirically in the original article describing network motifs in *E. coli,* which showed that bi-fan motifs are organised in dense overlapping regulons which consist of small numbers of TFs and operons that have particularly dense connectivity, and which also have few connections to the rest of the network [3]. Other work in E. coli has shown that clustering individual bi-fan motifs by overlap of any of their components leads to recovery of the network's largest fully connected component, and that a similar property can be observed for FFLs [16].

Many of the bi-fan arrays and the motifs within them can be attributed to the WGD event that occurred recently in the evolution of *Saccharomyces,* with the overwhelming majority of these structures arising from duplication of TFs. These represent a subset of the duplicative bi-fan arrays within the network, suggesting that many more of these network structures may also arise from divergent mechanisms of network evolution. It is possible that structural or sequence similarity could be used to detect more complex bi-fan architectures arising from ancient TF gene duplications. However, this is complicated by the rapid sequence divergence of TFs [15,17,35] and the potential for a particular network topology to be created by several alternative combinations of TF duplication and edge rewiring. It is clear, however, that the TFs arising from WGD have a larger number of shared targets and conserved network motif properties than more ancient duplicates. An outstanding question is whether this property is caused solely by the late occurrence of WGD in *Saccharomyces* or is also affected by the different effects of gene dosage in single-gene duplication and WGD events [36].

Although many bi-fan arrays originate from TF duplication, there is evidence that this topology also arises from environmental selection via the accumulation of DNA-binding motifs in promoter regions [22] or protein–protein interactions between TFs [8,24]. A mixture of these two effects is known to be a feature of mechanisms for combinatorial control of gene expression [26,37]. This article has also provided evidence that the cooperative binding of TFs to DNA is also likely to be involved in creating the functional divergence of duplicated TFs, as depicted in Figure 2C–2D. This mechanism may be particularly important for enabling increases in regulatory complexity to occur in unicellular organisms where redundant duplicate proteins cannot persist in the genome as a result of genetic drift [38], and consequently the fixation rate of single-gene duplications is very low [17].

The analysis of target genes indicates that the conservation of the TFs bound to duplicated promoters is related to the rate of sequence divergence of their associated genes, independently of molecular clock–based assumptions of the age of the duplication event [39,40]. This analysis also demonstrates that the *cis*-conservation is typically low and is restricted either to recent duplicates or the small number of genes that are stabilised by gene conversion [15,17]. Target gene duplication does not therefore make a substantial contribution to the formation of network motifs in the yeast TFN, contrary to other studies of *Saccharomyces* TFN evolution [11].

The rapid divergence in the promoters of duplicate genes is in agreement with other studies showing that gene expression evolves much more rapidly than an organism's gene content [12,13]. This result provides an explanation for a recent study of motif evolution [41], which found that the protein constituents of individual network motifs do not tend to co-occur across several very divergent yeast species. It was thus suggested that the motifs themselves are nonconserved and therefore not critical to the functionality of the network. However, the rapid *cis*-changes presented in Table 2 and the presence of positive selection toward motif formation suggest that the motif structures may be present in the comparison genomes, although their identity is likely to have changed on these relatively long time-scales. This is supported by the convergent evolution of similar network structures across diverse organisms, such as that observed between the human embryonic stem cell regulators SOX2, OCT4, and NANOG [42].

FFL motifs arise from a small number of regulatory interactions between TFs that form statistically significant bi-fan arrays. Our analysis indicates that there is likely to be positive environmental selection for the high/low-pass filtering properties of the FFL motif [3, 32] independently of the bi-fan array topology. As a result, FFL motifs could act as both a source and a consequence of duplicative bi-fan arrays in the course of network evolution. An outstanding question concerns the chronology of FFL formation, as it is not clear to what extent the existence of an FFL-like topology accelerates the accumulation of target genes or whether FFLs arise from existing bi-fan array structures, as depicted in Figure 3.

The static representation of the yeast TFN, representing a union of DNA-binding interactions across numerous environmental conditions, can be partitioned into modules that represent specific biological functions. Some structural families of DNA-binding proteins are not distributed uniformly across the network modules and are also involved in a larger number of bi-fan arrays with members of their own family. There are two potential causes for this observation. The WGD data indicates that TFs duplicated by WGD tend to occupy the same network module and share far more common targets than more ancient duplicates. It is therefore possible that proteins within a particular family underwent lineage-specific expansions more recently than other families. This appears to be the case for the *YAP* TFs, of which between two and three TF pairs originate from WGD [15,43]. The other possibility is that constraints on the diversity of binding sites available to a particular family of TFs [44,45] lead to a slower divergence of promoter binding, as exemplified by the GATA-binding family of Zinc-finger TFs.

In summary, the TFN contains many features that reflect the evolutionary history of the organism (i.e., divergent evolution), suggesting that its structure does not necessarily reflect an optimal “design” [46], and that evolutionary constraints contribute to both the modularity and network motifs that are present in the network. However, there is also strong evidence for the involvement of natural selection in the formation of network motifs beyond the neutral duplication–divergence model. The motif concept also provides a framework for understanding the mechanisms that have enabled increases in regulatory complexity to occur in a simple eukaryote, and which are also likely to apply to higher organisms.

## Methods

### Raw data.

The TFN was generated using the original gene-mapped ChIP-on-chip data from Harbison et al. [10]. The raw binding profiles were thresholded at a *p*-value of 10^−3^. TFs were classed as bound to an intergenic region if the binding profile was below the threshold in any of the assays carried out under alternative growth conditions. This included around 11,000 unique interactions between regulators and promoter regions.

### Network randomization procedures.

Randomization of the networks was carried out using modified versions of the two algorithms used in [3,8]. Both these methods ensure that the networks' degree distributions remain unchanged by fixing both *k_in_* and *k_out_* for each node [47] while randomly rewiring edges. One of the algorithms involves repeatedly swapping nonisomorphic pairs of directed edges until the network is sufficiently randomized. The second algorithm involves specifying a set of *in* and *out* stubs for each node. Directed edges are then added from each *out* stub to a randomly selected *in* stub while again preserving the networks' in- and out-degree distributions. The two algorithms for generating null networks were found to produce identical results, provided that a sufficient number of iterations were carried out in the edge-swapping algorithm.

### Organization of bi-fan motifs in directed networks.

The number of bi-fan motifs within the TFN, *f*_bi-fan_, can be rewritten in an alternative form, which suggests that this particular motif is, in general, associated with array structures such as that shown in Figure 1

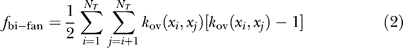
where the summations are over the *N_T_* TFs, or nodes with nonzero out-degrees, and where *k*(*x_i_, x_j_*) is the number of targets shared by TFs *x_i_* and *x_j_*. Equation 2 implies that for bi-fan motifs to be overrepresented in the network, there must be pairs of TFs (*x_i_, x_j_*) that have a greater number of shared targets than under an equivalent null model of the network.

The standard approaches to generating null network models [3,8,47] involve randomization of directed edges while preserving the in- and out-degree of each node. This null model provides an additional constraint on Equation 2

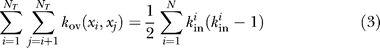
where *k^i^_in_* is the in-degree of node *i* and *N* is the total number of nodes in the network. Intuitively, Equation 3 represents the frequency of “mono-fans” in the network (i.e., two TFs binding to the same target). The left-hand side of Equation 3 represents the frequency of “mono-fans” in terms of the number of shared targets for each pair of TFs, which may vary in different randomizations of the network. The right-hand side represents this quantity in terms of the (fixed) in-degree sequence.

The constraint in Equation 3 indicates that a high degree of overlap for a subset of the TFs, required for overrepresentation of bi-fan motifs, implies a lower number of shared targets for other pairs of TFs. This suggests that bi-fan motifs are characteristic of networks with a modular or community structure [3,33].

### Detecting bi-fan arrays.

**Figure 8:**
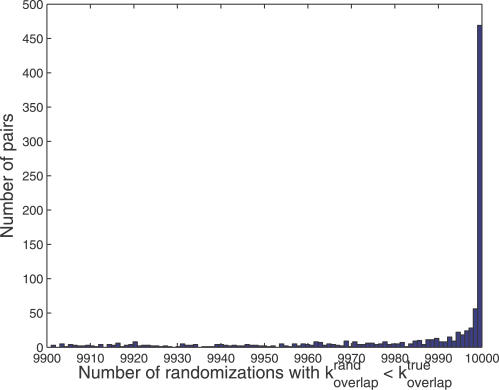
Histogram Representing Bootstrap-Estimated *p*-Values for Bi-Fan Array

Bi-fan arrays were identified by searching for pairs of TFs with a number of shared targets that exceeded the number found in 9,995 of the randomizations of the network. Figure 8 indicates the number of bi-fan arrays identified at the highest significance thresholds. Since there are a total of 176 TFs with *k_out_* ≠ 0 in the ChIP-on-chip dataset [10], there are a total of 1.54 × 10^4^ comparisons. A total of 595 arrays were recovered at this threshold, with an expected number of 15.4 for a random network.

The number of targets shared by pairs of TFs in the randomized networks is well approximated by a Poisson distribution, which was used to estimate *p*-values for the bi-fan arrays identified to be significant from the bootstrap estimates (see Text S1). A total of 442 of the bi-fan arrays were significant at the threshold, which is the stringent threshold used in further analyses. A total of 297 bi-fan arrays were found at the *p* < 0.05 threshold after a Bonferroni correction for the multiple hypotheses tested.

### Domain assignments.

The Pfam domain assignments were verified using the Saccharomyces Genome Database (http://www.yeastgenome.org), which also provided annotations for three additional TFs (*INO4p, XBP1p,* and *CUP1p*) that were missed by Pfam. The basic leucine zipper predictions were manually subdivided into the *YAP* and *AP-1* families using definitions from the literature [48]. The two largest families of TFs in yeast, the classic Zinc-finger and the Zn-Cys binuclear cluster domain, are short, ancient domains that typically form one of many contact points between the TF and DNA [49,50]. Consequently, the shared presence of these domain types is not necessarily indicative of recent divergence or similar DNA-binding specificity. These families were therefore subdivided using sequence clustering. The BLASTclust program was used with sequence identity set to 25% and the alignment length parameter set to 0.25. This procedure may result in more distant duplicates being missed but increases the statistical significance of any homologous bi-fan arrays identified from analysis of the yeast TFN (groupings can be found in Text S1).

### Statistical modelling of the formation of FFL arrays.

Several generalized linear models [31] were used to fit the probability of a regulatory interaction between a pair of TFs, *f*(*π_i_*), as a function of local network properties.

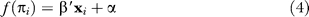
where **x**_i_ = [x_1_, x_2_, …, x_j_] is the vector of network properties, β and α are the parameters of the model, and *f*(·) is the link function. Several link functions, including linear, logistic, and log–log, were compared using the deviance and the Hosmer-Lemshow criterion [31]. The log–log model provided the best fit under both measures and was used to model the full set of network variables.

The initial set of variables were the out-degree of node *a*, *k^a^_out_*, the out-degree of node *b*, the number of targets shared by the pair of TFs, *k^ab^_array_*, the expected number of shared targets, and binary variables representing a feedback or autoregulatory interaction at node *a*, autoregulation at node *b* (*k^b^_auto_*), transcription regulation of node *a* by node *b*, homology, and genome duplication. Backward stepwise elimination was then used to remove uninformative variables (see Text S1 and Figures S3 and S4 for further details), and resulted in the following model,

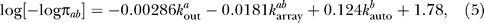
indicating that the probability of forming a regulatory interaction between TFs increases with the out-degree of node *a* and the number of targets shared by TFs *a* and *b*. Conversely, interactions are suppressed if the second TF *b* directly regulates its own transcription.

### Modularity in biological networks.

The modularity of the network is defined using the criterion *Q*, which is defined for undirected networks, but can be applied to the *Saccharomyces* TFN by considering each edge as undirected [33],

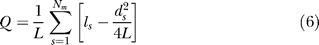
where the sum is over the number of identified modules, *N_m_*, *L* is the number of edges in the network, *l_s_* is the number of intramodule edges, and *d_s_* is the sum of the degrees of the nodes in module *s*. Intuitively, a cluster contributes a large Δ*Q* to the network's overall modularity if the number of intramodular connections is much larger than the number expected in an equivalent network with edges placed at random (a null model that corresponds exactly to the randomization procedures used in this article [47]).

The standard approach to module identification is to seek a partition of the network such that the modularity, Δ*Q*, is maximised. In this study, a spectral module detection algorithm [34] is used, which involves solving a series of eigenvector problems on a characteristic *modularity* matrix. The algorithm divides the network recursively into disjoint binary partitions until no further increase in the modularity is recovered. The division of the network can then be used to calculate the sensitivity of *Q* to the deletion of nodes from the network, Δ*Q*.

## Supporting Information

Figure S1 Frequency of Common Homology Relationships as Bi-Fan Arrays Are Added to the Network According to Their Statistical Significance The solid green curve represents common DNA-binding domains; the black curve, TFs originating from WGD; and the red curve, TFs that have a curated protein–protein interaction in the BioGrid database (http://www.thebiogrid.org). The dotted lines represent the expected frequencies under random addition of bi-fan arrays.
(22 KB EPS)

Figure S2 Frequency of Three-Node Bi-Fan Cliques Containing a Pair of WGD Duplicates as Three-Node Cliques Are Formed by Addition of Edges to the Network (18 KB EPS)

Figure S3 Likelihood Ratio of Regulator–Regulator Interactions as a Function of the Number of Shared Targets of a Pair of Transcription Regulators (9 KB EPS)

Figure S4 Likelihood Ratio of Regulator–Regulator Interactions as a Function of the Sum of the Out-Degrees of the Pair of Transcription Regulators (11 KB EPS)

Table S1 The Number of Proteins from Major Families of TF within the Yeast Proteome (29 KB DOC)

Table S2 Properties of TFs Originating from WGD in the Ancestor of S. cerevisiae
The *p*-values represent the probability of recovering more than the observed number of targets from a randomized replicate of the network.
(46 KB DOC)

Table S3 Fates of Duplicate TFs The columns represent, from left to right: bi-fan arrays participated in by each TF, the number of bi-fan arrays that are shared by the pair of TFs, the modules each TF is assigned to by the network clustering algorithm, and the sensitivity of the modularity parameter to deletion of each TF (Δ*Q*). The duplicate marked in bold is the putative orthologue (i.e., retains the majority of the ancestral functions).
(62 KB DOC)

Text S1 Supplementary Material (51 KB DOC)

## Abbreviations

ChIP: chromatin immunprecipitation
FFL: feed-forward loop
TF: transcription factor
TFN: TF network
WGD: whole-genome duplication
