# Supplementary material for: Evolutionary Models for Formation of Network Motifs and Modularity in the Saccharomyces Transcription Factor Network
Source: PLoS Comput Biol. 2007 Oct 26;3(10):e198. doi: 10.1371/journal.pcbi.0030198 (PMC2041975; doi:10.1371/journal.pcbi.0030198)
Supplement: Table S1 — (29 KB DOC) [file pcbi.0030198.st001.doc]

| Definition | Proteome | Harbison *et al.* |
| --- | --- | --- |
| Basic leucine zipper | 13 | 13 |
| Erythroid transcription factor GATA1 | 10 | 7 |
| Forkhead DNA-binding domain | 4 | 3 |
| Helix-loop-helix DNA binding domain | 8 | 8 |
| Heat-shock transcription factor | 5 | 5 |
| DNA-binding domain of Mlu1-box | 5 | 5 |
| (GCGC) binding protein MBP1 |  |  |
| Classic zinc finger, CH | 34 | 27 |
| Zn-Cys binuclear cluster domain | 54 | 41 |
| Whole Genome Duplication | 72 | 34 |
