# Supplementary material for: Evolutionary Models for Formation of Network Motifs and Modularity in the Saccharomyces Transcription Factor Network
Source: PLoS Comput Biol. 2007 Oct 26;3(10):e198. doi: 10.1371/journal.pcbi.0030198 (PMC2041975; doi:10.1371/journal.pcbi.0030198)
Supplement: Table S2 — The p-values represent the probability of recovering more than the observed number of targets from a randomized replicate of the network. (46 KB DOC) [file pcbi.0030198.st002.doc]

| Standard | k_out_ | Standard | k_out_ | Shared | Expected | *P*-value |
| --- | --- | --- | --- | --- | --- | --- |
| name |  | name |  | targets | targets | x10^-2^ |
| OAF1 | 61 | PIP2 | 36 | 29 | 1.09 | <0.01 |
| PDR3 | 21 | PDR1 | 95 | 0 | 0.95 | 62.83 |
| REB1 | 185 | YDR026C | 20 | 5 | 1.73 | 0.53 |
| SWI5 | 120 | ACE2 | 92 | 29 | 5.15 | <0.01 |
| MET32 | 75 | MET31 | 34 | 14 | 1.26 | <0.01 |
| YAP6 | 177 | CIN5 | 223 | 72 | 16.66 | <0.01 |
| CAD1 | 42 | YAP1 | 99 | 18 | 2.02 | <0.01 |
| YHP1 | 20 | YOX1 | 43 | 3 | 0.43 | 0.09 |
| STP1 | 79 | STP2 | 11 | 3 | 0.42 | 0.03 |
| ACA1 | 2 | CST6 | 26 | 0 | 0.03 | 2.76 |
| RCS1 | 347 | AFT2 | 168 | 41 | 24.02 | 0.01 |
| SKN7 | 216 | HMS2 | 44 | 10 | 4.34 | 0.20 |
| FKH1 | 142 | FKH2 | 169 | 54 | 10.69 | <0.01 |
| GZF3 | 57 | DAL80 | 52 | 19 | 1.45 | <0.01 |
| ZMS1 | 5 | YML081W | 8 | 0 | 0.02 | 2.31 |
| PHD1 | 123 | SOK2 | 73 | 35 | 4.25 | <0.01 |
| MSN4 | 188 | MSN2 | 164 | 101 | 13.37 | <0.01 |
