# Supplementary material for: Evolutionary Models for Formation of Network Motifs and Modularity in the Saccharomyces Transcription Factor Network
Source: PLoS Comput Biol. 2007 Oct 26;3(10):e198. doi: 10.1371/journal.pcbi.0030198 (PMC2041975; doi:10.1371/journal.pcbi.0030198)
Supplement: Table S3 — The columns represent, from left to right: bi-fan arrays participated in by each TF, the number of bi-fan arrays that are shared by the pair of TFs, the modules each TF is assigned to by the network clustering algorithm, and the sensitivity of the modularity parameter to deletion of each TF (ΔQ). The duplicate marked in bold is the putative orthologue (i.e., retains the majority of the ancestral functions). (62 KB DOC) [file pcbi.0030198.st003.doc]

| Protein a | Protein b | Bi-fans | Bi-fans | Shared | Module | Module | ΔQ_node_ | ΔQ_node_ |
| --- | --- | --- | --- | --- | --- | --- | --- | --- |
|  |  | a | b | Bi-fans | a | b | (x10^-3^) | (x10^-3^) |
| OAF1 | PIP2 | 2 | 2 | 2 | 3 | 3 | 1.403 | 1.370 |
| PDR3 | **PDR1** | 0 | 14 | 0 | 9 | 2 | 0.174 | 0.670 |
| REB1 | YDR026C | 1 | 0 | 0 | 9 | 9 | 4.553 | 0.263 |
| SWI5 | ACE2 | 4 | 7 | 2 | 11 | 11 | 0.063 | 0.284 |
| MET32 | MET31 | 4 | 2 | 2 | 3 | 3 | 1.703 | -0.466 |
| **YAP6** | CIN5 | 26 | 6 | 6 | 16 | 16 | 1.578 | 3.924 |
| CAD1 | **YAP1** | 2 | 8 | 2 | 5 | 18 | 0.287 | -1.362 |
| YHP1 | **YOX1** | 0 | 20 | 0 | 14 | 18 | 0.108 | -0.159 |
| **STP1** | STP2 | 12 | 1 | 0 | 1 | 2 | 0.820 | 0.191 |
| RCS1 | **AFT2** | 2 | 5 | 1 | 9 | 9 | 1.598 | -3.486 |
| **SKN7** | HMS2 | 9 | 4 | 1 | 12 | 12 | -4.565 | -0.497 |
| FKH1 | **FKH2** | 2 | 5 | 2 | 11 | 11 | 2.059 | 1.906 |
| GZF3 | DAL80 | 25 | 18 | 13 | 2 | 2 | 0.539 | 0.342 |
| PHD1 | SOK2 | 11 | 15 | 11 | 16 | 16 | 0.425 | 0.425 |
| MSN4 | MSN2 | 13 | 11 | 8 | 1 | 1 | -1.124 | 0.120 |
