# Supplementary material for: Evolutionary Models for Formation of Network Motifs and Modularity in the Saccharomyces Transcription Factor Network
Source: PLoS Comput Biol. 2007 Oct 26;3(10):e198. doi: 10.1371/journal.pcbi.0030198 (PMC2041975; doi:10.1371/journal.pcbi.0030198)
Supplement: Text S1 — (51 KB DOC) [file pcbi.0030198.sd001.doc]

Supplementary Material for “Evolutionary Models for Formation of Network Motifs and Modularity in the *Saccharomyces* Transcription Factor Network”

J. J. Ward and J. M. Thornton

## Data Quality Control

Transcription regulatory interactions were defined using the raw ChIP-on-chip data from [Harbison et al., 2004], which contains most representatives of the major structural families of DNA-binding proteins present in the yeast proteome (see Table S1). The Young lab estimated the false positive rates of their ChIP-on-chip protocol to be 6-10% and the false negative rate to be around 24%, at the threshold [Lee et al., 2002]. Although this increases the false positive rate from the 4% found by also requiring stringent conservation of consensus transcription factor binding sites in *sensu stricto* yeast species [Harbison et al., 2004], this additional criterion may also increase the false negative rate. This step was also taken to avoid introducing biases associated with the presence of *canonical* binding sites, such as the tendency of promoters under combinatorial regulation to contain binding sequences that are less clearly defined than those under the control of a single transcription factor [Bilu and Barkai, 2005]. The results were found to be insensitive to deletion of up to 30% of the edges in the transcription factor network (TFN), although the number of statistically significant bi-fan arrays was typically reduced (data not shown).

## Defining Common Evolutionary Origin

Table S2 shows properties of homologous transcription factors that were duplicated by WGD. All pairs where both components bind more than 10 targets form statistically significant bi-fan arrays, except for the pleiotropic drug resistance genes PDR1 and PDR3. This may represent an example of the rapid evolution of DNA-binding propensities for members of the Zn-Cys family of transcription factors [Vashee et al., 1993]. However, few of the promoters bound by PDR1 and PDR3 (only two in both cases) contain the experimentally derived consensus DNA-binding sites for these proteins [Christie et al., 2004]. There is also other evidence that these proteins co-bind DNA as heterodimers [Mamnun et al., 2002], so it cannot be ruled out that some aspect of the experimental protocol has not altered the DNA-binding properties of PDR1 and PDR3.

## Statistical Significance of Bi-fan Arrays involving Homologous Transcription Factors

We have described two potential mechanisms for the formation of bi-fan arrays: duplicative and accumulative. Common DNA-binding domains are potential signatures of duplicative bi-fan arrays, however, it is also possible that the proteins involved could have originated from an ancient duplication event, diverged and then subsequently accumulated common targets. Figure S1 indicates that there is a far higher probability of bi-fan arrays forming between transcription factors with a common evolutionary origin than expected if bi-fan arrays occur with equal probability between any two pairs of TF in the network. In particular, Figure S1 shows that WGD duplicates are expected to be involved in only 0.37 bi-fan arrays under the random model, thus providing evidence for the duplicative model of array formation. Figure S1 also shows that bi-fan arrays occur more frequently than expected between TFs that form a protein-protein interaction, thus supporting the alternative evolutionary models described in the main text.

## Computing the Statistical Significance of Non-Homologous Duplicative Bi-fan Arrays arising from WGD

The higher-order effects of gene duplication (Figure 3(C) from the main text) were identified by computing the frequency of cliques of three transcription factors that form bi-fan arrays with each other, and where two members were duplicated by WGD. The frequency of these features as bi-fan arrays are added to the network according to P-value, in addition to expected frequencies under random assignment of homology relationships is shown in Figure S2. In the randomization experiments 71% of replicates did not contain a single triplet containing a randomized WGD relationship.

## Statistical modelling the formation of feed-forward loop-forming interactions

The majority of feed-forward loops in the yeast TFN result from one or two direct regulatory interactions existing between transcription factors that form a statistically significant bi-fan array (Figure S3). However, the probability of forming such a regulatory interaction also increases with k_out_, as shown by Figure S4. The data points in Figures S3 and S4 were generated by constructing equally-populated bins, and fitting a linear least-squares curve to the mean likelihood ratio *L*=*P*(link|*X*)/*P*(link) score for each bin.

Generalized linear models [Dobson, 2002] were used to test whether these effects existed independently of each other, and also to test whether other network properties influence the probability of feed-forward loop formation. The generalized linear models of the probability for forming a regulator-regulator interaction were compared using the Hosmer-Lemshow criterion, which involves constructing equally-populated bins and computing a χ-squared statistic on the observed and expected counts of regulatory interactions [Dobson, 2002], and the deviance, *D*,

(1)

where y_i_ is a binary variable representing presence/absence of the regulatory interaction and π_i_ is the probability estimated by the model.

The log-log model provided the best fit under both measures and was used to model the full set of network variables, with uninformative variables removed by backward step-wise elimination. The influence of each variable on the probability of forming a regulator-regulator interaction was determined by computing the deviance of each model with a single variable removed. The model with highest deviance was then compared to the full model, and if the difference in the deviances was not statistically significant at the *P* < 0.05 level under a χ^2^(1) distribution, the variable was eliminated. This procedure was iterated until no further variables were removed.

## Network Modularization

The yeast TFN was partitioned recursively using the spectral module identification algorithm. Two small modules (containing 5 and 3 genes) that were unconnected to the rest of the *Saccharomyces* TFN were omitted. The functions of genes in each extant module were assigned using the EASE online web-tool [Dennis et al., 2003]. Full descriptions of the most statistically significant EASE terms are contained in the supplementary data.

Table S3 shows the properties of duplicated transcription factors in terms of network modules. The bold type indicates the duplicate that retains the majority of the ancestral functions (orthologue). The operational definition of function, used here, is the number of bi-fan interactions with other TFs, and assumes that loss (sub-functionalization) is the dominant source of functional divergence. Pairings are assigned as orthologue/paralogue where one TF is involved in fewer than half of the bi-fan arrays of its duplicate partner.

**References**

[Bilu and Barkai, 2005] Bilu, Y. and Barkai, N., 2005. The design of transcription-factor binding sites is affected by combinatorial regulation. *Genome Biol*, **6**(12):R103.

[Christie et al., 2004] Christie, K. R., Weng, S., Balakrishnan, R., Costanzo, M. C., Dolinski, K., Dwight, S. S., Engel, S. R., Feierbach, B., Fisk, D. G., Hirschman, J. E., *et al.*, 2004. Saccharomyces Genome Database (SGD) provides tools to identify and analyze sequences from Saccharomyces cerevisiae and related sequences from other organisms. *Nucleic Acids Res*, **32**(Database issue):311–314.

[Dennis et al., 2003] Dennis, G. J., Sherman, B. T., Hosack, D. A., Yang, J., Gao, W., Lane, H. C., and Lempicki, R. A., 2003. DAVID: Database for Annotation, Visualization, and Integrated Discovery. *Genome Biol*, **4**(5):P3.

[Dobson, 2002] Dobson, A. J., 2002. *An Introduction to Generalized Linear Models*. Chapman & Hall.

[Harbison et al., 2004] Harbison, C. T., Gordon, D. B., Lee, T. I., Rinaldi, N. J., Macisaac, K. D., Danford, T. W., Hannett, N. M., Tagne, J.-B., Reynolds, D. B., Yoo, J., *et al.*, 2004. Transcriptional regulatory code of a eukaryotic genome. *Nature*, **431**(7004):99–104.

[Lee et al., 2002] Lee, T. I., Rinaldi, N. J., Robert, F., Odom, D. T., Bar-Joseph, Z., Gerber, G. K., Hannett, N. M., Harbison, C. T., Thompson, C. M., Simon, I., *et al.*, 2002. Transcriptional regulatory networks in Saccharomyces cerevisiae. *Science*, **298**(5594):799–804.

[Mamnun et al., 2002] Mamnun, Y. M., Pandjaitan, R., Mahe, Y., Delahodde, A., and Kuchler, K., 2002. The yeast zinc finger regulators Pdr1p and Pdr3p control pleiotropic drug resistance (PDR) as homo- and heterodimers in vivo. *Mol Microbiol*, **46**(5):1429–1440.

[Stark et al., 2006] Stark, C., Breitkreutz, B.-J., Reguly, T., Boucher, L., Breitkreutz, A., and Tyers, M., 2006. BioGRID: a general repository for interaction datasets. *Nucleic Acids Res*, **34**(Database issue):535–539.

[Vashee et al., 1993] Vashee, S., Xu, H., Johnston, S. A., and Kodadek, T., 1993. How do "Zn2 cys6" proteins distinguish between similar upstream activation sites? Comparison of the DNA-binding specificity of the GAL4 protein in vitro and in vivo. *J Biol Chem*, **268**(33):24699–24706.
